# Leukemia stem cells: the root of chronic myeloid leukemia

**DOI:** 10.1007/s13238-015-0143-7

**Published:** 2015-03-10

**Authors:** Hong Zhou, Rongzhen Xu

**Affiliations:** 1 Key Laboratory of Cancer Prevention and Intervention, China National Ministry of Education, Department of Hematology, Zhejiang University, Hangzhou, 310009 China; 2 Cancer Institute, Second Affiliated Hospital, School of Medicine, Zhejiang University, Hangzhou, 310009 China

**Keywords:** chronic myeloid leukemia (CML), leukemia stem cells (LSCs), tyrosine kinase inhibitors (TKIs), CaMKII-γ, molecular switch

## Abstract

Chronic myeloid leukemia (CML) is a clonal myeloproliferative disorder characterized by a chromosome translocation that generates the Bcr-Abl oncogene encoding a constitutive kinase activity. Despite remarkable success in controlling CML at chronic phase by Bcr-Abl tyrosine kinase inhibitors (TKIs), a significant proportion of CML patients treated with TKIs develop drug resistance due to the inability of TKIs to kill leukemia stem cells (LSCs) that are responsible for initiation, drug resistance, and relapse of CML. Therefore, there is an urgent need for more potent and safer therapies against leukemia stem cells for curing CML. A number of LSC-associated targets and corresponding signaling pathways, including CaMKII-γ, a critical molecular switch for co-activating multiple LSC-associated signaling pathways, have been identified over the past decades and various small inhibitors targeting LSC are also under development. Increasing evidence shows that leukemia stem cells are the root of CML and targeting LSC may offer a curable treatment option for CML patients. This review summarizes the molecular biology of LSC and its-associated targets, and the potential clinical application in chronic myeloid leukemia.

## INTRODUCTION

It is well established that cancers depend on a small population of “cancer stem cells” (CSCs) for maintaining their uncontrolled growth. Early insight into the molecular pathogenesis of cancer stemmed from the discovery of activating oncogenes, such as Bcr-Abl oncogene and its constitutively active protein tyrosine kinase, in CML (NOWELL and HUNGERFORD, 1960; Rowley, 1973; Druker, 2008). The Bcr-Abl oncogenic tyrosine kinase is responsible for initiating and maintaining the leukemia phenotype of CML cells. This oncoprotein is also responsible for the phosphorylation, activation and dysregulation of intracellular signaling proteins that regulate the survival and growth of progenitor cells in the bone marrow (Tauchi et al., 1994; Kabarowski and Witte, 2000). Although tyrosine kinase inhibitors (TKIs) for Bcr-Abl oncoprotein revolutionized the treatment of CML at chronic phase (CML-CP), 25% of TKI-naive patients and 50%–90% of patients with TKI-resistance contain leukemic clones expressing TKI-resistant Bcr-Abl kinase mutants (Slupianek et al., 2013). Studies have shown that a number of factors, including activation of critical signal transduction pathways, cell cycle alterations, abnormal epigenetic events and aberrant microenvironment from the hematopoietic niche, contribute to the enhanced survival and continued growth of CML stem/progenitor cells. This review will focus on the molecular biology of LSC and its-associated targets, and the potential clinical application in chronic myeloid leukemia.

## LEUKEMIA STEM CELLS OF CHRONIC MYELOID LEUKEMIA

Stem cells are defined as functional rather than morphological or any other physical feature. The current definition of stem cell remains the same as it established by McCulloch and Till, which are characterized by three distinct properties: self-renewal, differentiation capability, and proliferative capacity (Molofsky et al., 2005). These properties make stem cells to produce and maintain a diverse and specialized group of cells. In addition to maintaining their undifferentiated state by self-renewal, stem cells also generate progenitors that differentiate to form cells of multiple lineages upon successive divisions.

CML has long been considered as a stem cell disease, but detailed characteristics of its stem cells have not been fully elucidated to date. Because CML has the propensity to evolve from a chronic phase to accelerated phase and finally to blast crisis phase, one can pinpoint the phase and developmental stage at which mutations arise. Hence, CML is an important paradigm for understanding both genetic and epigenetic events that drive aberrant stem and progenitor cell differentiation, self-renewal and survival during both early and advanced phases of disease (Lowe and Sherr, 2003). *In vitro* studies using long-term culture-initiating cell (LTC-IC) assays showed the presence of pluripotent stem cells of malignant origin in patients with CML (Chen et al., 1994). The majority of CML progenitors were found to have a higher proliferative capacity compared to normal progenitors, suggesting that most CML progenitors were actively cycling (Eaves et al., 1998).

The concept that cancer/leukemia stem cells (CSCs/LSCs) are responsible for initiation, drug resistance, and relapse of cancers has inflamed this area of research and the importance of CSCs has been demonstrated in a variety of tumors (Morrison et al., 1995; Weissman, 2000; Al-Hajj et al., 2003). In CML and other malignancies, studies have shown that LSCs are able to self-renew, which leads to therapeutic resistance and disease progression (Olsson et al., 2014).

A model for leukemogenesis shows that the malignant transformation of normal hematopoietic stem/precursor cells would give rise to LSCs (Dash et al., 2002; Zhao et al., 2004; Strathdee et al., 2007), which retains the key characteristics of self-renewal and proliferative capacity but do not differentiate to mature cells. Because current therapies for leukemia are designed based on the general biological properties of malignant blast cells with proliferation potential, whereas LSCs are frequently in a quiescent state. Thus, current strategies do not effectively eliminate the LSCs as well as the disease (Holyoake et al., 1999).

### Quiescence of leukemia stem cells

Although the precise molecular mechanism of LSC-mediated resistance to current therapies has not been fully elucidated, one critical factor might be the quiescence of LSC that allows this population cells to evade the targeting by current therapies. In CML, abnormal tyrosine kinase-directed phosphorylation and mislocalization of cell cycle proteins have been implicated in deregulation of the cell cycle in Bcr-Abl expressing cells, which means that CML quiescent LSCs are TKI resistant and represent a Bcr-Abl kinase-independent disease reservoir (Cramer et al., 2008). Leukemia stem cells, particularly those in a quiescent state, are highly resistant to current chemotherapies and targeted therapies, resulting in disease relapse (Ito et al., 2008; Kaminska et al., 2008). In addition, signaling molecules involved in cell survival and self-renewal, which are the two critical characteristics of quiescent LSC, have been linked to key regulators of the cell cycle.

Studies have revealed that LSCs residing in the bone marrow niche are dormant and resistant to traditional chemotherapies. Specific signals from the surrounding stromal cells might promote LSCs cell cycle arrest and allow them to persist even during treatment with TKI therapies. Imatinib mesylate (IM), the first drug designed to target the Bcr-Abl kinase, induces hematologic and cytogenetic remissions in the majority of CML patients at chronic phase, however, the Bcr-Abl kinase domain mutations portend a greater risk of loss of complete cytogenetic remission (CCR) (Molofsky et al., 2005). Ultimately, regardless of greatly reduced mortality rates with Bcr-Abl targeted therapy, a significant proportion of patients are expected to develop TKI resistance driven by quiescent LSCs, which may be a reservoir for disease progression to blast crisis. Several studies demonstrate that a quiescent population of CML stem cells (CD34^+^CD38^–^CD45RA^–^CD71^–^HLA^–^DR^low^) with Bcr-Abl kinase domain mutations, detectable prior to initiation of imatinib therapy, gives rise to leukemia cells that persist because they are inherently resistant to imatinib (Sorel et al., 2004; Molofsky et al., 2005; Barnes and Melo, 2006; Jiang et al., 2007; Jorgensen et al., 2007; Niemann et al., 2007; Wodarz, 2008; Olsson et al., 2014). This may be attributable in part to quiescent LSCs residing in the protective niches that acquire additional mutations over time. In addition, quiescent CD34^+^ progenitors at chronic phase increases the expression levels of chemokines associated with stem cell mobilization (Dierks et al., 2008). Also, several oncogenic transcription factors that regulate cell-fate decision in HSCs and have been implicated in myeloid leukemia, such as MEIS1 and HOXA9, were found to be overexpressed in blast-crisis CML (Jamieson et al., 2004a, b). HOXA9 overexpression leads to the transformation of primary bone marrow cells through specific collaboration with MEIS1 (Kroon E et al., 1998). It has been suggested that HOXA9 forms ternary complexes with PBX2 and MEIS1 in myeloid leukemic cells (Shen WF et al., 1999). These results indicate that the ternary complex, including HOXA9, plays an important role in inducing leukemia, Cooperative activation of MEIS1 and HOXA9 perturbs myeloid differentiation and eventually leads myeloid progenitors to leukemia.

### Self-renewal and survival of leukemia stem cells

Deregulation of programmed cell death or apoptosis allows cancer stem cells to propagate even as they detach from the niche and accumulate genetic mutations. In CML, resistance to apoptosis begins with Bcr-Abl, and notably, the final consequence of Bcr-Abl inhibition with imatinib is induction of apoptosis (Holyoake et al., 1999). CML stem cell resistance to apoptosis involves the aberrant expression of the Bcl-2 family of apoptosis-regulatory proteins, including anti-apoptotic members, such as Bcl-2 and Mcl-1, and pro-apoptotic members, such as Bad and Bim (Bedi et al., 1994; Cramer et al., 2008). In addition, activation of the Wnt/β-catenin pathway leads to increased c-myc expression and ultimately results in upregulation of Bcl-2 family proteins. However, Neviani et al. reported that protein phosphatase 2A (PP2A)-activating drugs (PADs) can markedly reduce the survival and self-renewal of CML quiescent HSCs, but not normal quiescent HSCs through Bcr-Abl kinase-independent and PP2A-mediated inhibition of JAK2 and β-catenin (Neviani et al., 2013). In addition, autocrine TNF-alpha production supports the survival of CML stem and progenitor cell and enhances their proliferation (Gallipoli et al., 2013).

Several important apoptosis proteins are also highly regulated by the PI3K/Akt pathway (Cramer et al., 2008). Akt directly inhibits Bad and Bax and modulates the activity of transcription factors such as those from the NF-κB and FoxO families (Ito et al., 2008; Kaminska et al., 2008). NF-κB protein activation leads to its nuclear localization, where it then activates the transcription of a number of pro-survival molecules including Bcl-2, Bcl-xl, and various caspase inhibitors (Cramer et al., 2008). The importance of these transcription factors in CML has been highlighted by the fact that NF-κB is activated in transgenic models of CML and that Bcr-Abl can activate NF-κB (Kaminska et al., 2008). FoxO transcription factors, on the other hand, are targeted for proteolysis via Akt-mediated signaling (Khorashad et al., 2008). These factors normally induce the transcription of pro-death molecules including Bim and FasL. In CML, Bcr-Abl causes constitutive repression of FoxO3a by continued activation of Akt, leading to another mechanism of Bcr-Abl-mediated apoptotic resistance (Khorashad et al., 2008; Scheller et al., 2013). Finally, Akt can inhibit GSK3β activity. Thus, the Akt pathway is involved in apoptosis regulation via regulation of GSK3β in CML. Although many survival pathways are upregulated in CML, they are also active in normal stem cells. Thus, revealing the differential expression profiles of survival-related genes between normal hematopoietic stem cells and leukemia stem cells will be critical for designing therapies that selectively eradicate CML stem cells while sparing normal stem cells.

### Aberrant differentiation of leukemia stem cells

In CML progenitors at blast crisis, the characteristic block of myeloid differentiation is resulted from Bcr-Abl and MAPK (ERK1/2)-induced hnRNP-E2 RNA-binding protein-mediated suppression of C/EBPα expression (Chang et al., 2007). The imatinib and nilotinib resistance, which is derived in part from autocrine granulocyte-macrophage colony-stimulating factor (GM-CSF) secretion in response to adaptive JAK2/STAT5 signaling in granulocyte-macrophage progenitors (GMPs), may be overcome by JAK2 inhibitor (Wang et al., 2007; Gallipoli et al., 2014). In addition to cell autocrine effects, study results from a tetracycline-off mouse model revealed that osteopontin, an essential component of the stem cell niche, was upregulated in Bcr-Abl positive cells (Jamieson et al., 2004a, b). Similarly, osteopontin levels were higher in the serum of patients with CML and contributed to maintain the malignant clones at extramedullary sites (Jamieson et al., 2004a, b). A genetically defined mouse model of blast crisis CML demonstrated that coincident overexpression of genes that skew differentiation, such as Bcr-Abl and NUP98/HoxA, also resulted in the production of an imatinib-resistant LSC population (Dash et al., 2002; Neering et al., 2007). Together these data suggest that the block of differentiation and the enhancement of self-renewal in CML stem cells may be a critical step of disease progression.

## SIGNALING PATHWAYS OF LEUKEMIA STEM CELLS

Self-renewal is an essential property of stem cells, but the aberrant activation of self-renewal related signaling pathways has been recognized as a hallmark of cancer (Lowe and Sherr, 2003; Jamieson et al., 2004a, b). Wnt genes encode small secreted proteins existing in all animal genomes, and Wnt signaling is involved in virtually every aspect of embryonic development and also controls homeostatic self-renewal in a number of adult tissues. Studies have shown that the developmental pathways, such as the Wnt signaling pathway and the polycomb-group protein Bmi1, are involved in the regulation and expansion of LSCs during CML blast crisis (Chen et al., 1994; Reya et al., 2001; Saudy et al., 2014). The Wnt pathway, which is critical for HSC self-renewal and interaction with bone marrow niche, was found to be activated during progression to advanced phase in CML patients at blast crisis (Lowe and Sherr, 2003). Self-renewal capacity is normally absent in GMP, but some studies reveal that inappropriate activation of the Wnt pathway in GMPs endows these cells with self-renewal capacity, as measured by generation of replatable myeloid colonies (Lowe and Sherr,^2003^), suggesting that these GMPs acquires a key property of a leukemia stem cell. β-Catenin is also essential for survival of LCSs. In some cases β-catenin activation is associated with deregulation of GSK3β, an essential negative regulator of β-catenin, in both stem and progenitor cells, while other patients exhibit defective expression of another negative regulator of the pathway-axin 2 (Liu et al., 2005; Quaiser et al., 2006). The progression to myeloid blast crisis can be averted in a β-catenin knockout mouse model of CML (Sengupta and Banerjee, 2007).

Aberrant activation of another self-renewal program, Sonic hedgehog, was shown to induce expansion of LSCs in a mouse model of CML, while another study reported that there might be a cross-talk between sonic hedgehog, Wnt, and notch pathways in CML (Williams et al., 2008; Hu et al., 2009). The Hedgehog (Hh) signaling pathway is a developmental pathway that has been shown to play a role in primitive and adult hematopoiesis (Morrison et al., 1995). The Hh pathway activity is required for maintaining the normal and leukemia stem cells, and raising the possibility that the drug resistance and disease recurrence might be avoided by targeting the essential stem cell maintenance pathway. Strathdee et al. reported that the Hh signaling was activated in LSCs and differentiated hematopoietic cells through the upregulation of Smo, an essential component of the Hh pathway (Strathdee et al., 2007). Constitutively activation of Smo increased CML stem cell number and accelerated disease (Zhao et al., 2009), while the loss of Smo impaired HSC renewal (Strathdee et al., 2007).

**Figure 1:**
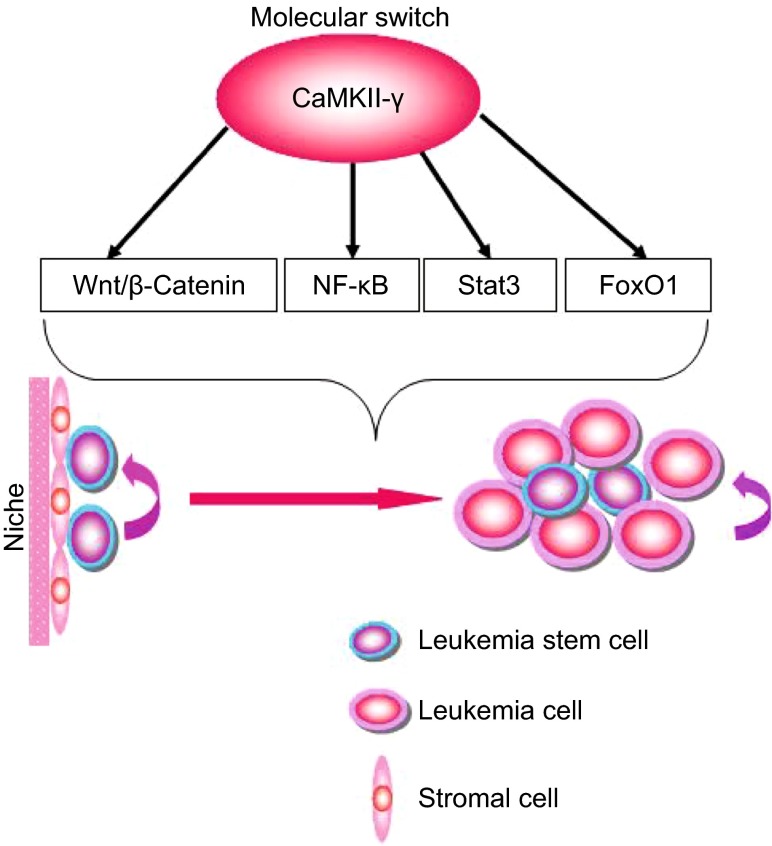
CaMKII-γ promotes self-renewal of leukemia stem cells via co-activating multiple signaling pathways

CaMKII comprises a family of closely related kinases with four isoforms (α, β, γ, and δ). Among these four isoforms, CaMKII-γ has been shown to be the major one overexpressed in myeloid cells and its potential role as a regulation of leukemia (Si et al., 2007), inhibition of CaMKII activity correlated with enhanced apoptosis (Hojabrpour et al., 2012). Also, CaMKII-γ regulates many fundamental cellular functions and has been implicated in the pathogenesis of several diseases (Si et al., 2007; Si and Collins, 2008). Our study found that both total and phosphor CaMKII-γ proteins were highly expressed in the CD34^+^/CD38^−^ CML LSCs, but low in CD34^−^ CML cells, CD34^+^ HSCs from healthy cord bloods and normal blood cells, suggesting that CaMKII-γ might play a critical role in the survival and proliferation of CML LSCs. CaMKII-γ is a specific and critical target of berbamine for its anti-leukemia activity and berbamine selectively binds to CaMKII-γ by targeting ATP binding pocket (Gu et al., 2012). Most importantly, CaMKII-γ is a critical molecular switch of multiple cancer-related signaling pathways (Fig. 1), such as NF-κB (Hughes et al., 2001; Gu et al., 2012), Wnt/β-catenin (Si and Collins, 2008; Gu et al., 2012), ERK1/2, FoxO1, AKT, and Stat3 signaling pathways (Hughes et al., 2001; Marganski et al., 2005; Ang et al., 2007; Si et al., 2007; Liu et al., 2008; Bouallegue et al., 2009; Timmins et al., 2009; Gu et al., 2012).

Recently, Gerber et al. identified 97 genes that were differentially expressed in CML versus normal stem and progenitor cells by performing genome-wide transcriptome analysis of highly refined CML and normal stem and progenitor cell populations using exon microarrays. Further analyses of the LSCs revealed dysregulation of normal cellular processes; downregulation of pro-differentiation and TGF-beta/BMP signaling pathways; upregulation of oxidative metabolism and DNA repair pathways; and activation of inflammatory cytokines (Gerber et al., 2013). These data greatly contribute to understanding the molecular changes of LSCs and developing novel therapies for eradicating LSCs and even achieving curable effect of CML.

## LEUKEMIA STEM CELL-RELATED TRANSCRIPTION FACTORS

Progression of CML is associated with transcription factor-induced aberrant lineage priming of stem and progenitor cells. The role of transcription factor deregulation in the differentiation of CML stem cells was demonstrated in a transgenic mouse model with deficiency of a transcription factor, Jun B. Jun B deficiency led to the development of a CML-like disease with a propensity for myeloid differentiation in which serial transplantation potential existed only at the level of HSC (Passegue et al., 2001; Chu et al., 2005). Moreover, GM-CSF-mediated survival and proliferation of Jun B-deficient GMP was associated with anti-apoptotic proteins Bcl-2 and Bcl-x, and cell cycle regulators p16^ink4a^ and c-Jun (Chu et al., 2005). The transcriptional repressor Bmi1, which is normally restricted to the stem-cell compartment, was found to be overexpressed in the aggressive forms of CML that progress to blast crisis within 3 years and during advanced phases of this disease (Chang et al., 2007).

The E-twenty six (ets) transcription factor GA binding protein (GABP) is a tetrameric transcription factor complex that contains GABPα and GABPβ proteins. Deletion of GABPα induced cell cycle arrest and profound loss of hematopoietic progenitor cells in bone marrow (Yang et al., 2013). Recently, Yang et al. identified the serine-threonine kinase protein kinase D2 (PRKD2) as a potential effector of GABP in HSCs through bioinformatic screen. PRKD2 can rescue the growth of GABPα-null Bcr-Abl-expressing cells whereas inhibiting PRKD2 expression decreased cell cycling (Yang et al., 2013). These data indicate that GABP is essential for the cell cycle entry of HSC and CML development and may offer a potential therapeutic target for CML.

GATA-2, an important myeloid transcription factor, has been proven to be upregulated in CML CD34^+^ cells, whereas stem cell fate determination by the large family of Hox genes is negatively regulated by Bmi1 (Cao et al., 2005; Mohty et al., 2007). Transcription factors HoxA9 and HoxB4 are better known for their transforming potential via enhancing HSC self-renewal and abnormal myelopoiesis upon upregulation (Cao et al., 2005; Strathdee et al., 2007). Downregulation of Hox genes, such as HoxA5, impairs myelopoiesis, suggesting differentiation block at the level of hematopoietic stem cells. Interestingly, hypermethylation-mediated inactivation of HoxA4 and HoxA5 was found in 34% of CD34^+^ cells from patients with chronic phase CML whereas it was observed in up to 90% of patients with CD34^+^ CML in blast crisis (Strathdee et al., 2007). Moreover, this hypermethylation-mediated inactivation of HoxA4 and HoxA5 was associated with a poor prognosis in other myeloid and lymphoid malignancies and might be a biomarker of more severe disease (Strathdee et al., 2007).

## EPIGENETIC EVENTS IN LEUKEMIA STEM CELLS

Epigenetic alterations include DNA methylation, histone modifications, and miRNA in the permanent changes of gene expression, which controls the leukemia phenotype. Among these, DNA methylation and histone modification play an important role in leukemogenesis (Esteller, 2008; Jones et al., 2002). For instance, aberrant DNA methylation could silence the expression of tumor suppressor genes in leukemia whereas overexpression of the histone methyltransferase, EZH2, a subunit of the polycomb group repressive complex 2 (PRC2) promotes oncogenesis (Momparler et al., 2012).

SIRT1 deacetylase is a multifunctional protein and can potentially regulate the acetylation of several transcription factors, including p53 (Luo et al., 2001), Ku70, and FoxOs (Brooks and Gu, 2009). Recent studies demonstrate that SIRT1 deacetylase not only promotes acquisition of genetic mutations for drug resistance in CML cells but also play a critical role in maintaining the survival of CML LSCs (Wang et al., 2013; Li et al., 2012). SIRT1 activation promotes resistance of CML stem cells to tyrosine kinase inhibitors and acquisition of BCR-ABL mutations for acquired resistance. Consistently, SIRT1 was expressed at higher levels in human CML CD34^+^ cells than in normal CD34^+^ cells. Moreover, inhibition of SIRT1 increased apoptosis in LSC of chronic phase and blast crisis CML and reduced their growth *in vitro* and *in vivo*. The inhibitory effects of SIRT1 targeting on CML cells depend on p53 expression and acetylation. Importantly, SIRT1 inhibition had less of an effect on proliferation and apoptosis of normal CD34^+^ cells, suggesting that SIRT1 might be a new target for eliminating CML cancer stem cells. In addition, SIRT1 inhibition in combination with BCR-ABL tyrosine kinase inhibitors might be a novel approach to eliminate leukemic stem cells and residual disease in CML.

Alternative pre-mRNA splicing (AS) is an epigenetic process that greatly diversifies the repertoire of the transcriptome (Adamia et al., 2013). “Splicing programs” typically react to individual changes with considerable effects in cell proliferation, cell survival, and apoptosis, although the changes in these individual splicing events are small. Evaluation of AS events in CML can be used to identify new disease markers and sensitive targets in order to overcome the current limits of small molecule inhibitors for the treatment of patients with CML.

## CHEMOKINES AND MICROENVIRONMENT OF LEUKEMIA STEM CELLS

Chemokine is a large protein family that can be divided into subfamilies on the basis of structural motifs. An important property of chemokines is its leukocyte chemotaxis. Chemokines regulate their biological effects via interactions with a family of 7-transmembrane G protein-coupled receptors. Chemokines, such as stromal-derived factor-1 (SDF-1/CXCL12) secreted by stromal cells, attracts cancer cells by acting its cognate receptor CXCR4 (Peng et al., 2012). Previous studies indicated that chemokines, which were expressed on hematopoietic and nonhematopoietic tumor cells, were reported to mediate chemotaxis of CD34^+^ stem cells, and to play a critical role in the homing and retention of these cells in the microenvironment of bone marrow (Bleul et al., 1996; Kortesidis et al., 2005). In addition, SDF-1 was found to function as both a chemoattractant and as a modulator of cellular growth/survival (Naiyer et al., 1999; Cashman et al., 2002; Liu et al., 2011)

It is unclear why under certain conditions this may promote uncontrolled proliferation, while in other cases cell quiescence may be favored. However, it is possible that the cues from the microenvironment might be involved. The studies support important, new concepts regarding the contribution of leukemia-induced alterations in the BM microenvironment to a selective growth advantage to leukemic compared with normal long-term hematopoietic stem cell (LTHSC) and have relevance to our understanding of the response and resistance to TKI at the organismal level (Zhang et al., 2012). Also, evidence provides new insight into the potential contribution of the microenvironment to the initiation and progression of myeloid disorders and leukemia, and may provide a unique area for the development of combination therapeutic strategies aimed at eradicating the resistant LSC population in CML (Hobbs et al., 1998; Liotta and Kohn, 2001). Signals from the microenvironment have a profound influence on the maintenance and/or progression of hematopoietic cancers.

## TARGETING OF LEUKEMIA STEM CELLS

**Figure 2:**
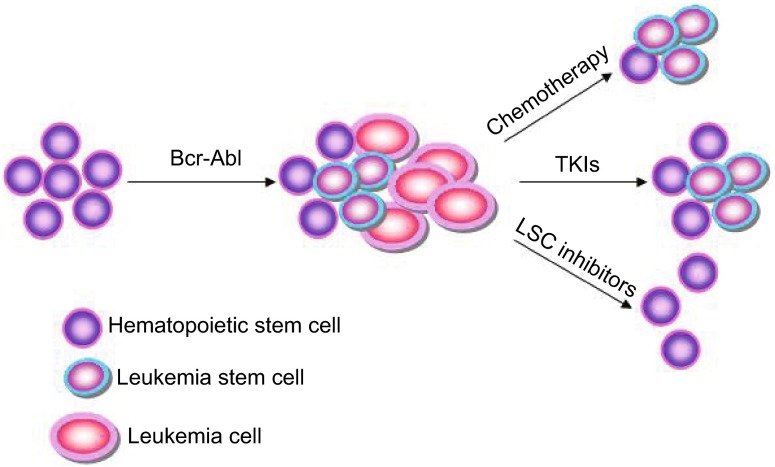
Model of different therapies for chronic myeloid leukemia. Hemotherapy kills both leukemia cells and normal hematopoietic stem cells. TKIs selectively kill leukemia cells but not leukemia stem cells. LSC inhibitors selectively kill leukemia stem cells

Despite the curable effect for CML by allogeneic hematopoietic cell transplantation, high transplant-related morbidity and mortality has greatly reduced its use since the development of molecularly targeted therapy (Karanes et al., 2008). Currently, there are three kinds of therapeutic drugs for CML: traditional chemotherapy agents, TKIs, and LSC inhibitors, and their working models are shown in Fig. 2. Before the development of Bcr-Abl-targeted therapy, treatment of CML patients at chronic phase is focused primarily on achieving hematologic control, and cytogenetic remission is rare. Moreover, most patients will progress to blast crisis within a few years. Currently, TKIs, which are potent inhibitors of Bcr-Abl protein kinases, c-Kit and the platelet-derived growth factor receptor (Valk-Lingbeek et al., 2004; Slupianek et al., 2013), are widely used for treatment of CML due to their highly efficient control of CML at chronic phase. However, These TKIs are unable to kill CML stem cells (Graham et al., 2002). A large number of studies have demonstrated that these TKIs can readily kill most CML cells but only display cytostatic effect on the primitive CML LSCs (Holtz et al., 2002). Consistent with these data, CD34^+^ stem cells from CML patients with complete cytogenetic responses were found to be of malignant origin with the capacity to give rise to CML-BC (Bhatia et al., 2003). These findings indicate that a reservoir of CML LSCs is still maintained in patients who do not show evidence of disease.

The leukemia stem cells in patients with CML are well known to be resistant to conventional chemotherapy and may also be relatively resistant to Bcr-Abl-targeted drugs. Studies have demonstrated the persistence of quiescent CML stem cells that are impervious to Bcr-Abl inhibitor therapy and contribute to disease progression (Holyoake et al., 1999; Chu et al., 2005; Barnes and Melo, 2006; Copland et al., 2006; Jorgensen et al., 2007). The precise sequence of molecular events leading to recalcitrance to Bcr-Abl inhibitor therapy as well as the cellular framework in which they occur has not been elucidated completely. As the properties of leukemia stem cells become better defined, it is possible to evaluate current therapeutic strategies correctly. Identification of therapeutic targets, which are involved in the survival of CML LSCs such as bcl-2; the self-renewal such as sonic Hh, bmi-1, notch, and Wnt; and the block differentiation such as JAK2/STAT5 activation and HOX gene hypermethylation, will contribute to target LSCs effectively using combinations of small molecule inhibitors and demethylating agents. (Guzman et al., 2005; Neviani et al., 2005; Guzman et al., 2007; Mohty et al., 2008; Bhattacharyya et al., 2009).

Several bcl-2 family member inhibitors are currently under development, JAK2/STAT5 inhibitors are currently being tested in clinical trials and self-renewal pathway inhibitors such as gamma-secretase inhibitors have been used clinically. Several Wnt and sonic hedge-hog inhibitors, which are being evaluated in preclinical models, show promise clinically for eradicating committed progenitors that have aberrantly gained self-renewal capacity. In addition, implementation of strategies to augment both innate and adaptive immune responses against CML progenitors may accelerate disease eradication (Rezvani et al., 2007; Yong et al., 2008).

The eukaryotic translation initiation factor 4E (eIF4E) is a potent oncogene elevated in an estimated 30% of human cancers (Culjkovic et al., 2009; Siddiqui et al., 2012), and the eIF4E protein provides the critical interface between mRNA, recruitment of eIF4A and eIF4G, and the 40S ribosomal subunits. The activity of eIF4F is generally regulated by altering the phosphorylation state of the eIF4E component. Increased phosphorylation of eIF4E correlates with enhanced translation in cells stimulated with mitogens, growth factors, or serum (Kaspar et al., 1990; Marino et al., 1989). Recently, studies showed that over-phosphoeylation of eukaryotic translation initiation factor 4E (eIF4E) led to increased β-catenin protein synthesis, whereas MAP kinase interacting serine/threonine kinase (MNK) kinase-dependent eIF4E phosphorylation at serine 209 is required for nuclear translocation and activation of β-catenin. These findings suggest that inhibiting phosphorylation of eIF4E may be therapeutically useful in CML-BC (Lim et al., 2013).

RAD51 (Rec A homolog of *E. coli*) is a polymorphic gene and one of the central proteins in homologous recombination-DNA-double-stand breaks (HR-DNA-DSB) repair pathway, which is vital in maintaining genetic stability within a cell (Hamdy et al., 2011). RAD51 recombinase activity plays a critical role for cancer cell proliferation and survival, and often contributes to drug-resistance, therefore been proposed as an alternative and supplementary strategy for cancer treatment. IBR2, a novel small molecule that inhibits cancer cell growth and induces apoptosis by inducing proteasome-mediated RAD51 protein degradation, significantly prolonged animal survival in a murine imatinib-resistant CML model bearing the T315I Bcr-Abl mutation. Moreover, IBR2 effectively inhibits the proliferation of CD34^+^ progenitor cells from CML patients, suggesting that small molecule inhibitors of RAD51 may provide a novel class of broad-spectrum therapeutics for difficult-to-treat cancers (Zhu et al., 2013).

A better understanding of the molecular biology of leukemia stem cells will lead to designing more effective therapies for CML. The challenge includes identification of differences between leukemia stem cells and their normal stem cell counterparts, and demonstration of complete control of leukemia by targeting these cancer stem cells. Recent advances in these areas have identified several novel target candidates that represent important avenues for future therapeutic approaches aimed at selectively eradicating the LSC population while sparing normal hematopoietic progenitors in patients suffering from chronic myeloid leukemia.

## ABBREVIATIONS

CCR: complete cytogenetic remission
CML: chronic myeloid leukemia
CML-CP: CML at chronic phase
CSC: cancer stem cell
eIF4Ε: eukaryotic translation initiation factor 4E
GABP: GA binding protein
GM-CSF: granulocyte–macrophage colony-stimulating factor
GMP: granulocyte–macrophage progenitor
Hh: Hedgehog
HR-DNA-DSB: homologous recombination-DNA-double-stand breaks
IM: imatinib mesylate
LSC: leukemia stem cells
LTC-IC: long-term culture-initiating cell
LTHSC: long-term hematopoietic stem cell
MNK: MAP kinase interacting serine/threonine kinase
PAD: PP2A-activating drug
PP2A: protein phosphatase 2A
PRC: polycomb group repressive complex
SDF-1: stromal-derived factor-1
TKI: tyrosine kinase inhibitors
